# EctD-mediated biotransformation of the chemical chaperone ectoine into hydroxyectoine and its mechanosensitive channel-independent excretion

**DOI:** 10.1186/s12934-016-0525-4

**Published:** 2016-07-20

**Authors:** Laura Czech, Nadine Stöveken, Erhard Bremer

**Affiliations:** Laboratory for Microbiology, Department of Biology, Philipps-University at Marburg, 35043 Marburg, Germany; LOEWE Center for Synthetic Microbiology, Philipps-University Marburg at Marburg, 35043 Marburg, Germany; Laboratory for Microbiology, Department of Biology, Philipps-University at Marburg, Karl-von-Frisch-Str. 8, 35043 Marburg, Germany

**Keywords:** Osmostress protectants, Compatible solutes, Ectoines, Heterologous production, Dioxygenase, Transporters, Efflux

## Abstract

**Background:**

Ectoine and its derivative 5-hydroxyectoine are cytoprotectants widely synthesized by microorganisms as a defense against the detrimental effects of high osmolarity on cellular physiology and growth. Both ectoines possess the ability to preserve the functionality of proteins, macromolecular complexes, and even entire cells, attributes that led to their description as chemical chaperones. As a consequence, there is growing interest in using ectoines for biotechnological purposes, in skin care, and in medical applications. 5-Hydroxyectoine is synthesized from ectoine through a region- and stereo-specific hydroxylation reaction mediated by the EctD enzyme, a member of the non-heme-containing iron(II) and 2-oxoglutarate-dependent dioxygenases. This chemical modification endows the newly formed 5-hydroxyectoine with either superior or different stress- protecting and stabilizing properties. Microorganisms producing 5-hydroxyectoine typically contain a mixture of both ectoines. We aimed to establish a recombinant microbial cell factory where 5-hydroxyectoine is (i) produced in highly purified form, and (ii) secreted into the growth medium.

**Results:**

We used an *Escherichia coli* strain (FF4169) defective in the synthesis of the osmostress protectant trehalose as the chassis for our recombinant cell factory. We expressed in this strain a plasmid-encoded *ectD* gene from *Pseudomonas stutzeri* A1501 under the control of the anhydrotetracycline-inducible *tet* promoter. We chose the ectoine hydroxylase from *P. stutzeri* A1501 for our cell factory after a careful comparison of the in vivo performance of seven different EctD proteins. In the final set-up of the cell factory, ectoine was provided to salt-stressed cultures of strain FF4169 (pMP41; *ectD*^+^). Ectoine was imported into the cells via the osmotically inducible ProP and ProU transport systems, intracellularly converted to 5-hydroxyectoine, which was then almost quantitatively secreted into the growth medium. Experiments with an *E. coli* mutant lacking all currently known mechanosensitive channels (MscL, MscS, MscK, MscM) revealed that the release of 5-hydroxyectoine under osmotic steady-state conditions occurred independently of these microbial safety valves. In shake-flask experiments, 2.13 g l^−1^ ectoine (15 mM) was completely converted into 5-hydroxyectoine within 24 h.

**Conclusions:**

We describe here a recombinant *E. coli* cell factory for the production and secretion of the chemical chaperone 5-hydroxyectoine free from contaminating ectoine.

**Electronic supplementary material:**

The online version of this article (doi:10.1186/s12934-016-0525-4) contains supplementary material, which is available to authorized users.

## Background

To balance the osmotic gradient across their cytoplasmic membrane and to maintain turgor, many microorganisms produce large amounts of organic osmolytes when they face high-osmolarity environments [1, 2]. These types of highly water soluble compounds are fully compliant with cellular physiology [3–5] and can therefore be accumulated to exceedingly high intracellular levels; they are generally referred to as compatible solutes [6]. In addition to their well-studied function as water-attracting osmolytes [7–9], compatible solutes also serve as chemical chaperones [10, 11]. They promote the stability and correct folding of proteins and macromolecular assemblies, preserve the integrity of membranes, and positively influence the functionality of nucleic acids [5, 12–16]. They exert these beneficial properties not only in vitro but also in vivo [11, 17, 18].

Ectoine [(*S*)-2-methyl-1,4,5,6-tetrahydropyrimidine-4-carboxylic acid] and its derivative 5-hydroxyectoine [(4*S*,5*S*)-5-hydroxy-2-methyl-1,4,5,6-tetrahydropyrimidine-4-carboxylic acid] are such compatible solutes [19, 20]. Many *Bacteria* and a few *Archae*a synthesize them in response to osmotic stress [21, 22]. Synthesis of ectoine proceeds from l-aspartate-β-semialdehyde [23, 24], and it is catalyzed by the sequential actions of l-2,4-diaminobutyrate transaminase (EctB; EC 2.6.1.76), 2,4-diaminobutyrate acetyltransferase (EctA; EC 2.3.1.178), and ectoine synthase (EctC; EC 4.2.1.108) [23, 25].

**Fig. 1:**
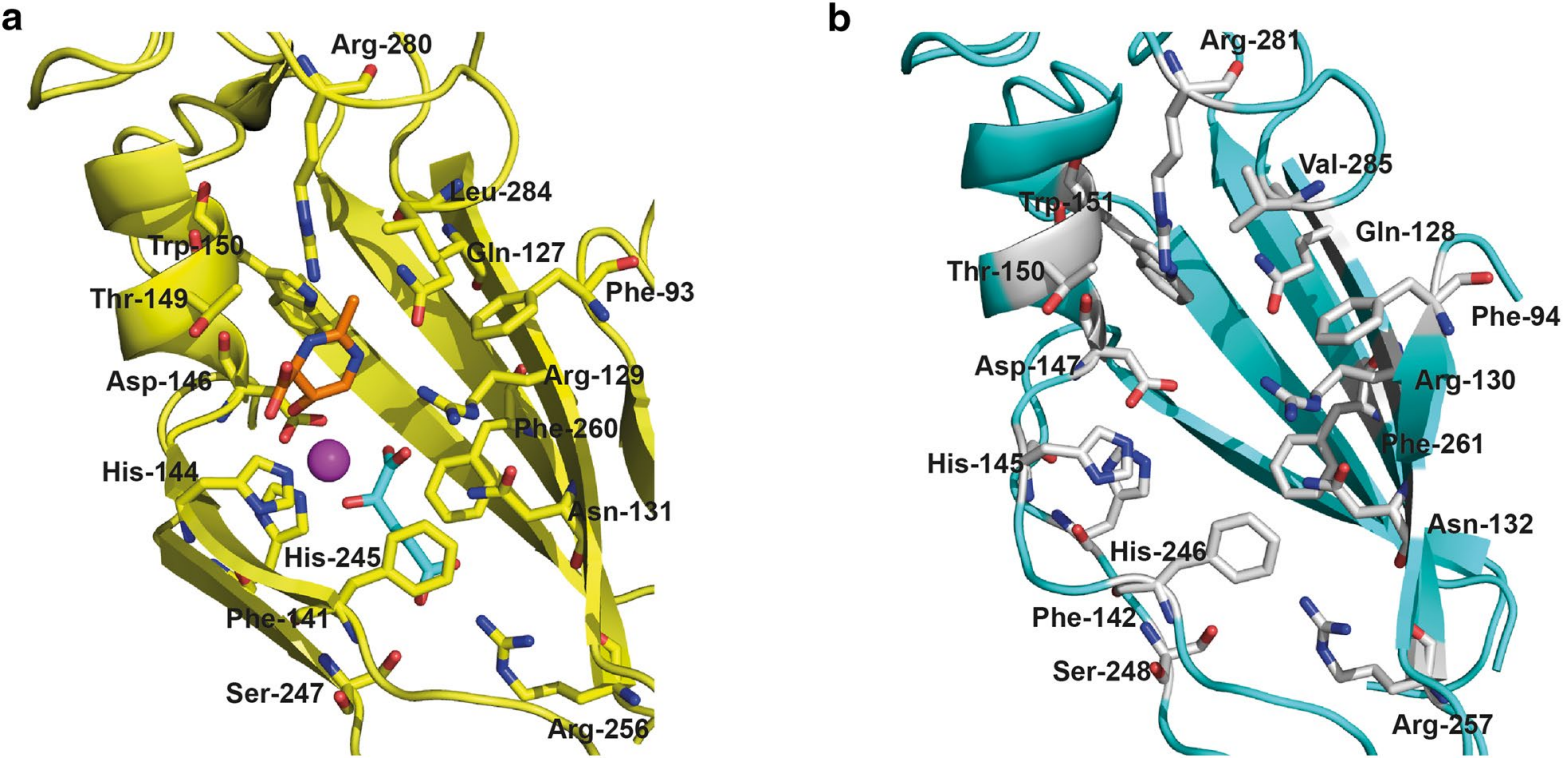
Architecture of the active site of the ectoine hydroxylase from *S. alaskensis* and in silico model of the EctD protein from *P. stutzeri* A1501. **a** Crystal structure of the ectoine hydroxylase from *S. alaskensis* containing the iron catalyst (*purple sphere*), the co-substrate 2-oxoglutarate (*blue sticks*) and the reaction product 5-hydroxyectoine (*orange sticks*) bound in the active side of the enzyme (PDB accession code: 4Q5O). Amino acids of the *S. alaskensis* EctD protein involved in ligand binding [27] are represented as *sticks*. **b** in silico model of the ectoine hydroxylase from *P. stutzeri* A1501 that is based on the crystal structure of the *S. alaskensis* EctD protein was build with the SWISS model web server (https://swissmodel.expasy.org/) [101]. Amino acids of the active site of the *P. stutzeri* A1501 EctD protein predicted to be involved ligand binding are shown as *grey sticks*

5-Hydroxyectoine is directly formed from ectoine through a position- and stereo-specific modification [20, 26], a reaction carried out by the ectoine hydroxylase (EctD; EC 1.14.11) [26–29]. The hydroxylation reaction catalyzed by EctD proceeds in an O_2_-dependent fashion, relies on a mononuclear iron center, uses 2-oxoglutarate as the co-substrate and also yields the side-products CO_2_ and succinate [26, 27, 29–31]. The ectoine hydroxylase [21, 22, 26–29] is a member of the non-heme-containing iron(II) and 2-oxoglutarate-dependent dioxygenases [32–35]. Ectoine hydroxylases are closely related in their amino acid sequence and can be distinguished from other members of non-heme-containing iron(II) and 2-oxoglutarate-dependent dioxygenases through the presence of a highly conserved signature sequence [22, 29, 31]. The EctD consensus sequence not only contains residues involved in iron, 2-oxoglutarate, and ectoine/hydroxyectoine binding, but also serves an important architectural role for the structuring of the cupin barrel [27, 29, 31]. A crystal structure of the ectoine hydroxylase from the cold-adapted bacterium *Sphingopyxis alaskensis* in complex with the catalytically important iron, the co-substrate 2-oxoglutarate, and the reaction product 5-hydroxyectoine has been reported [27]. EctD is a homo-dimer both in solution and in the crystal [22, 27, 29], and a view into the active site of this enzyme is shown in Fig. 1a.

5-Hydroxyectoine possesses stress-protective properties that go well beyond its role in osmotic adjustment. Indeed, synthesis of 5-hydroxyectoine often increases in stationary growth phase [26, 36, 37], a condition that confronts microbial cells with considerable physiological challenges [38]. Increased production of 5-hydroxyectoine is also induced in response to increased growth temperature [36, 37, 39], suggesting an in vivo protein stabilizing function at temperatures sub-optimal for growth. The hydroxylation of ectoine endows the newly formed 5-hydroxyectoine with enhanced, or additional, function-preserving properties [28, 36, 40, 41]. For example, 5-hydroxyectoine is a superior desiccation protectant [40, 42], a property that is dependent on its ability to form glasses [41]. Furthermore, 5-hydroxyectoine possesses a superior stabilizing effect on the structural organization of lipid monolayer and bilayer membranes, an attribute that likely stems from the fact that its –OH group partly replaces the water molecules lost from the hydration shell of lipids [16]. Both ectoines have different effects on the melting temperature of DNA; ectoine lowers the melting temperature while 5-hydroxyectoine increases it [43]. As a result, addition of 5-hydroxyectoine to the hybridization solution significantly improves the quality of DNA-microarrays [44]. Most widely noted are the often better protein-stabilizing properties of 5-hydroxyectoine in comparison with its direct biosynthetic precursor ectoine [13, 45–47].

The stabilizing and function-preserving attributes of ectoines led to various practical applications [45, 48, 49] and to the development of an industrial scale production process harnessing the highly salt tolerant bacterium *Halomonas elongata* as a microbial cell factory [50]. In this production process, *H. elongata* is grown in media of elevated salinity to trigger high-level ectoine production. These cells are then subjected to a strong osmotic downshift that leads to the transient opening of mechanosensitive channels [51, 52] and the concomitant release of the newly formed ectoine into the culture supernatant from which it can readily be purified [45, 48]. This ectoine-production scheme has been fashionably coined “*bacterial milking*” [53].

In addition to the assessment of natural ectoine- and 5-hydroxyectoine-producing microorganisms for practical purposes [45, 48, 49], there have been recently considerable efforts to use recombinant DNA techniques to design synthetic cell factories for these compounds. These efforts include plasmid-based expression systems for the ectoine/5-hydroxyectoine biosynthetic genes (*ectABCD*) placed either under the transcriptional control of the natural osmotically inducible *ect* promoter or of various synthetically inducible promoters [39, 54–56]. Typically, ectoines produced in this way are retained inside the cells. However, excretion of ectoine or 5-hydroxyectoine has been repeatedly observed when the expression of ectoine/5-hydroxyectoine biosynthetic genes was engineered in non-natural ectoine producers, for instance the Gram-negative bacterium *Escherichia coli*, the Gram-positive bacterium *Corynebacterium glutamicum*, and the yeast *Hansenula polymorpha* [55, 57–59].

With notable exceptions [37, 39], those microorganisms capable of synthesizing 5-hydroxyectoine typically produce naturally a mixture of ectoine and 5-hydroxyectoine, a fact that requires additional purification steps during down-stream processing to obtain pure 5-hydroxyectoine for practical applications [45, 48, 49]. Since 5-hydroxyectoine has a number of interesting attributes, we wondered whether it might be possible to design a synthetic microbial cell factory that could take up ectoine, quantitatively convert it into 5-hydroxyectoine, and secrete almost the entire product into the growth medium. Here we report the implementation of a bacterial cell factory with these desired characteristics.

## Results

### Basic design of the hydroxyectoine cell factory

**Fig. 2:**
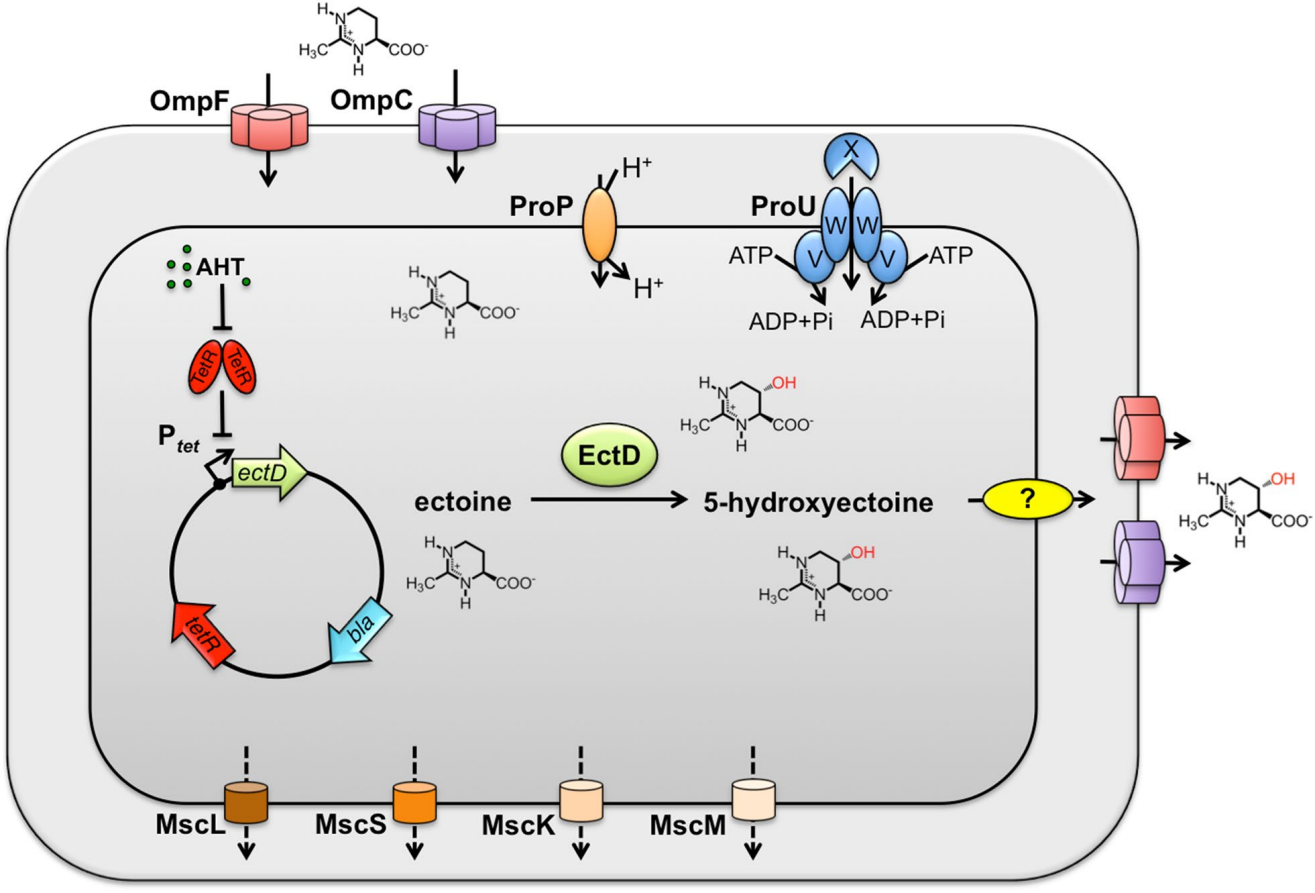
Schematic representation of the microbial cell factory for the heterologous production of 5-hydroxyectoine. Ectoine available in the medium diffuses passively across the outer membrane of *E. coli* via the OmpC and OmpF porins into the periplasm. It is then actively transported across the cytoplasmic membrane into the *E. coli* cell via the two osmotically inducible compatible solute transporters, ProP and ProU. The gene (*ectD*) for the ectoine hydroxylase (EctD) is expressed from the TetR-controlled and AHT-responsive *tet* promoter present on the expression plasmid. The newly synthesized EctD enzyme hydroxylates ectoine to 5-hydroxyectoine, most of which is then released into the growth medium via an unknown mechanism. Various types of mechanosensitive channels (MscL, MscS, MscK, MscM) operating in *E. coli* are indicated

*Escherichia coli* cannot synthesize ectoine but it can import it via the osmotically inducible ProP and ProU osmostress protectant uptake systems [60, 61]. ProP is a proton/solute symporter and a member of the major facilitator (MFS) superfamily [62], whereas ProU belongs to the multi-component ABC-type of transport systems [63, 64]. Ectoine is likely to permeate across the *E. coli* outer membrane by diffusion through the general porins OmpC and OmpF (Fig. 2), as has been shown for the osmostress protectant glycine betaine [65]. We therefore speculated that the heterologous expression of an ectoine hydroxylase gene (*ectD*) [26, 27] in *E. coli* will probably result in the conversion of the imported ectoine into 5-hydroxyectoine and its possible excretion from the recombinant cell factory (Fig. 2). The latter assumption is based on the observation that the synthetic production of ectoine in *E. coli* leads to the excretion of the newly synthesized compound [55, 57].

Trehalose is the only compatible solute that *E. coli* synthesizes de novo as a stress protectant when it is challenged by high osmolarity [66]. To avoid a contamination of the desired 5-hydroxyectoine with trehalose, we used a strain [FF4169; *otsA*::Tn*10*] [66] which is deficient in trehalose synthesis as our cell factory. For the heterologous production of the ectoine hydroxylase (EctD), we used a set of plasmids in which a particular *ectD* gene is expressed from the strong and tightly regulated *tet* promoter present on the backbone of the used cloning vectors pASK-IBA3 and pASG-IBA3 (IBA, Göttingen, Germany). The *tet* promoter is negatively controlled by the TetR repressor (Fig. 2) whose DNA-binding activity can be abrogated by adding the synthetic inducer anhydrotetracycline (AHT) to the growth medium.

The *ectD* genes used in our study were derived from various extremophilic microorganisms (*H. elongata*, *S. alaskensis*, *Virgibacillus salexigens, Pseudomonas stutzeri*, *Paenibacillus lautus*, *Alkalilimnicola ehrlichii*) [22], and the marine archaeon *Nitrosopumilus maritimus* [21]. Some of these cloned genes were directly derived form chromosomal DNA of the donor microorganisms (*H. elongata*, *S. alaskensis*, *P. stutzeri*, *V. salexigens*), whereas others (*P. lautus*, *A. ehrlichii*, *N. maritimus*) are synthetic, codon-optimized versions of *ectD* genes [21, 22]. The proteins are of similar length and molecular mass, predicted pI and they exhibit a degree of amino acid sequence identity (relative to that of the *P. stutzeri* EctD protein; see below) between about 53 and 41 % (Additional files 1, 2). The residues involved in iron, co-substrate, and substrate binding by the ectoine hydroxylase [22, 27, 29, 31] are fully conserved in the EctD proteins assessed in our study (Additional file 2).

### In vivo benchmarking of ectoine hydroxylases

The kinetic parameters of the seven ectoine hydroxylases employed for our experiments have been obtained with the purified EctD proteins under carefully optimized in vitro conditions for each enzyme. They all possess similar enzyme activities with *K*_*m*_ values ranging from about 6 to 10 mM for the substrate ectoine and *V*_*max*_ values ranging between about 1 and 7 U mg^−1^ of protein [21, 22]. These data are summarized in the Additional file 1. Since in vitro data on the kinetic properties of the EctD enzymes might not necessarily reflect their in vivo performance in a heterologous host bacterium, we benchmarked the ability of the seven enzymes to convert ectoine to 5-hydroxyectoine in the above described chassis strain to identify the best suited EctD enzyme for its application in the cell factory.

**Fig. 3:**
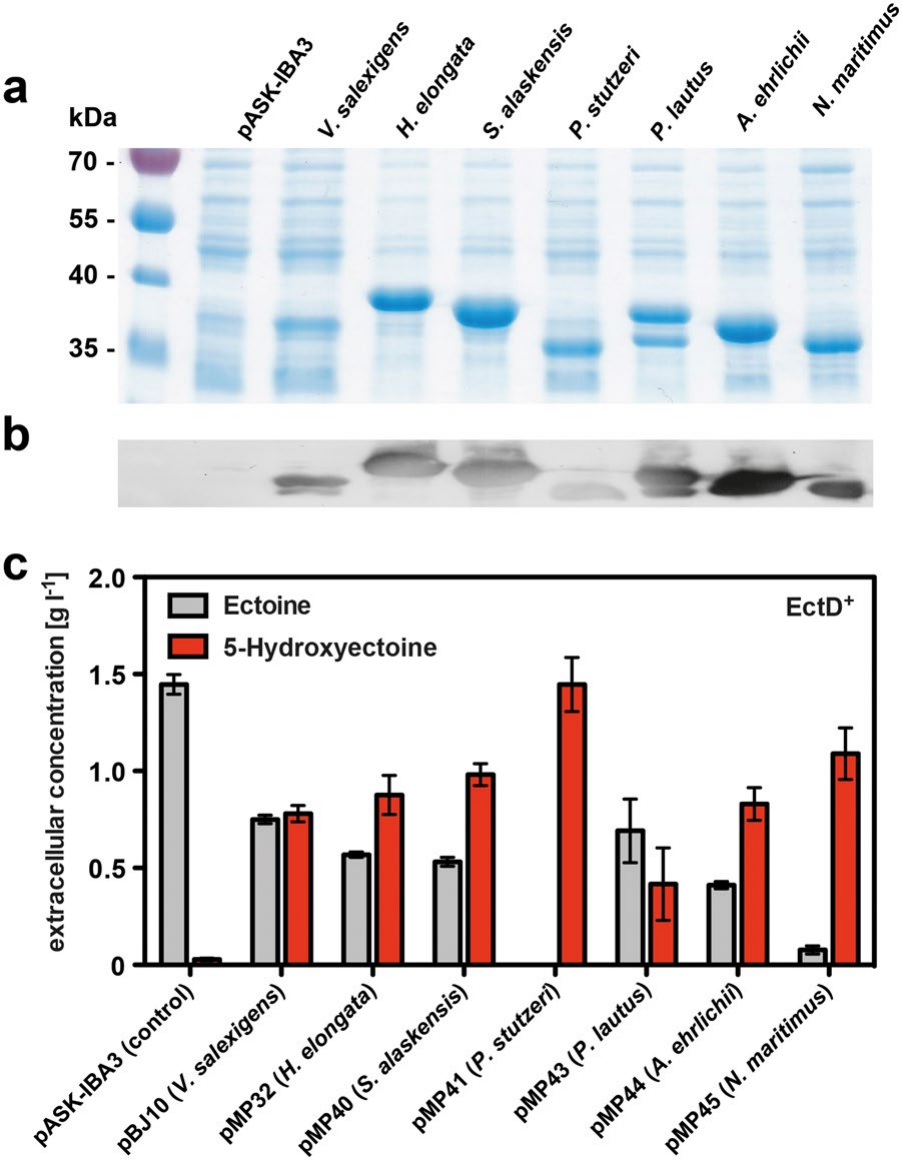
Conversion of ectoine to 5-hydroxyectoine by EctD proteins from different microorganisms. **a** Overproduction of EctD proteins originating from *V. salexigens*, *H. elongata, S. alaskensis, P. stutzeri, P. lautus, A. ehrlichii*, and *N. maritimus* was performed in the *E. coli* strain FF4169, and total cellular extracts were analyzed on an 15 % SDS–polyacrylamide gel. **b** Production of EctD-*Strep*-tag II proteins from *V. salexigens*, *H. elongata, S. alaskensis, P. stutzeri, P. lautus, A. ehrlichii*, and *N. maritimus* was confirmed by Western blot analysis using a monoclonal antibody directed against the *Strep*-tag II affinity peptide (SA-WSHPQFEK) attached to the various EctD proteins [21, 22]. **c** The ectoine (*grey*) and 5-hydroxyectoine (*red*) content of the culture supernatants were assessed via HPLC analysis. Strain FF4169 harboring an *ectD* ^+^ plasmid was grown in MMA in shake flasks in the presence of ampicillin and 0.4 M NaCl that had been supplemented by the addition of 10 mM ectoine. The values given for the ectoine and 5-hydroxyectoine content are the means and standard deviations of two independently grown cultures

To this end, we expressed seven plasmid-encoded *ectD* genes in the *E. coli* strain FF4169 under high-saline conditions (with 0.4 M NaCl) and in the presence of 10 mM ectoine (this corresponds to 1.42 g l^−1^) in the growth medium. We then analyzed the amount of the EctD protein by inspecting whole cell extracts applied to SDS polyacrylamide gel electrophoresis (Fig. 3a), and we also measured the ectoine and 5-hydroxyectoine content of the growth medium in cultures that had been propagated for 24 h (Fig. 3c). With the exception of the EctD protein from *V. salexigens*, all ectoine hydroxylases were produced in substantial amounts (Fig. 3a) and reacted with an antibody directed against the *Strep*-tag II affinity peptide that had been attached to the corresponding proteins to allow their purification [21, 22] in a Western blot experiment (Fig. 3b). Despite the close match of the various EctD proteins with respect to their calculated molecular mass (Additional file 1), substantial variations in their electrophoretical mobility was observed and signs of proteolysis was noted in two of the EctD proteins (Fig. 3a, b).

There were substantial differences in the ability of the tested ectoine hydroxylases to convert ectoine into 5-hydroxyectoine (Fig. 3c). In most cases a mixture of the added ectoine and the newly produced 5-hydroxyectoine were observed in the culture supernatants. There were two striking exceptions where almost all of the provided ectoine had been converted into 5-hydroxyectoine; these were the ectoine hydroxylases from *P. stutzeri* A1501 [22] and from the archaeon *N. maritimus* [21] (Fig. 3c), leading to the production of 1.57 ± 0.08 and 1.23 ± 0.05 g l^−1^ 5-hydroxyectoine, respectively, from the originally 1.42 g l^−1^ ectoine (this corresponds to 10 mM) provided to the cells. Based upon these initial experiments, we chose the ectoine hydroxylase [22] from the plant root-associated bacterium *P. stutzeri* A1501 [67] for the following experiments. A homology model of the *P. stutzeri* EctD enzyme assessed by us showed that this protein adopts in all likelihood a three-dimensional structure matching that of the crystalized EctD protein from *S. alaskensis* (Fig. 1a, b).

### Optimization of the parameters for the 5-hydroxyectoine cell factory

**Fig. 4:**
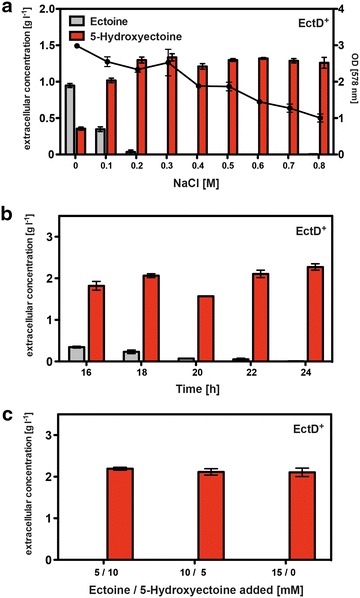
Optimization of the cell factory for 5-hydroxyectoine production. **a** The *E. coli* strain FF4169 carrying plasmid pMP41 (the *ectD* gene from *P. stutzeri* A1501) was cultivated for 24 h in MMA containing ampicillin and 0.4 M NaCl in the presence of 10 mM ectoine (growth of the cultures is plotted on the *right Y-axis*) (*black dots*). The ectoine (*grey*) and 5-hydroxyectoine (*red*) content of the supernatants were examined via HPLC analysis (plotted on *left Y-axis*). **b** Samples from cultures of strain FF4169 (pMP41) propagated in MMA with 0.4 M NaCl and 15 mM ectoine were taken after 16, 18, 20, 22 and 24 h of growth and the ectoine (*grey*) and 5-hydroxyectoine (*red*) content of the supernatant was analyzed by HPLC. **c** The *E. coli* strain FF4169 (pMP41) was grown for 24 h in MMA containing ampicillin and 0.4 M NaCl in the presence of different ratios of ectoine and 5-hydroxyectoine. The amounts of both compounds in the supernatant were quantified by HPLC analysis; ectoine (*grey*) and 5-hydroxyectoine (*red*) bars. All values shown in parts (**a**), (**b**), and (**c**) are the means and standard deviations of at least two independent cultures

The ProP and ProU ectoine uptake systems in *E. coli* are osmotically regulated both at the level of *proP* and *proU* transcription and at the level of their transport activity [60, 62, 68]. As a consequence, increased osmolarity will be a key determinant for the efficient uptake of ectoine by the FF4169 chassis strain. We therefore assessed the influence of increased sustained salinity for the biotransformation of ectoine by growing the cells in media in which the NaCl concentration was increased in a finely tuned manner. In keeping with the osmotic control of the ProP and ProU systems, substantial amounts of ectoine remained in the medium of the cultures that received no additional NaCl (Fig. 4a). Moderate increases in salinity reduced the amount of ectoine remaining in the medium and resulted in an increased 5-hydroxyectoine production (Fig. 4a). When the growth medium contained more than 0.3 M NaCl, there was complete uptake of the provided 10 mM ectoine. It was converted into 5-hydroxyectoine, and the newly formed 5-hydroxyectoine was excreted into the growth medium (Fig. 4a). Notably, this level of salinity had only a modest effect on the growth yield of the cultures (Fig. 4a), an important parameter that needed to be considered for the final set-up of the cell factory.

**Fig. 5:**
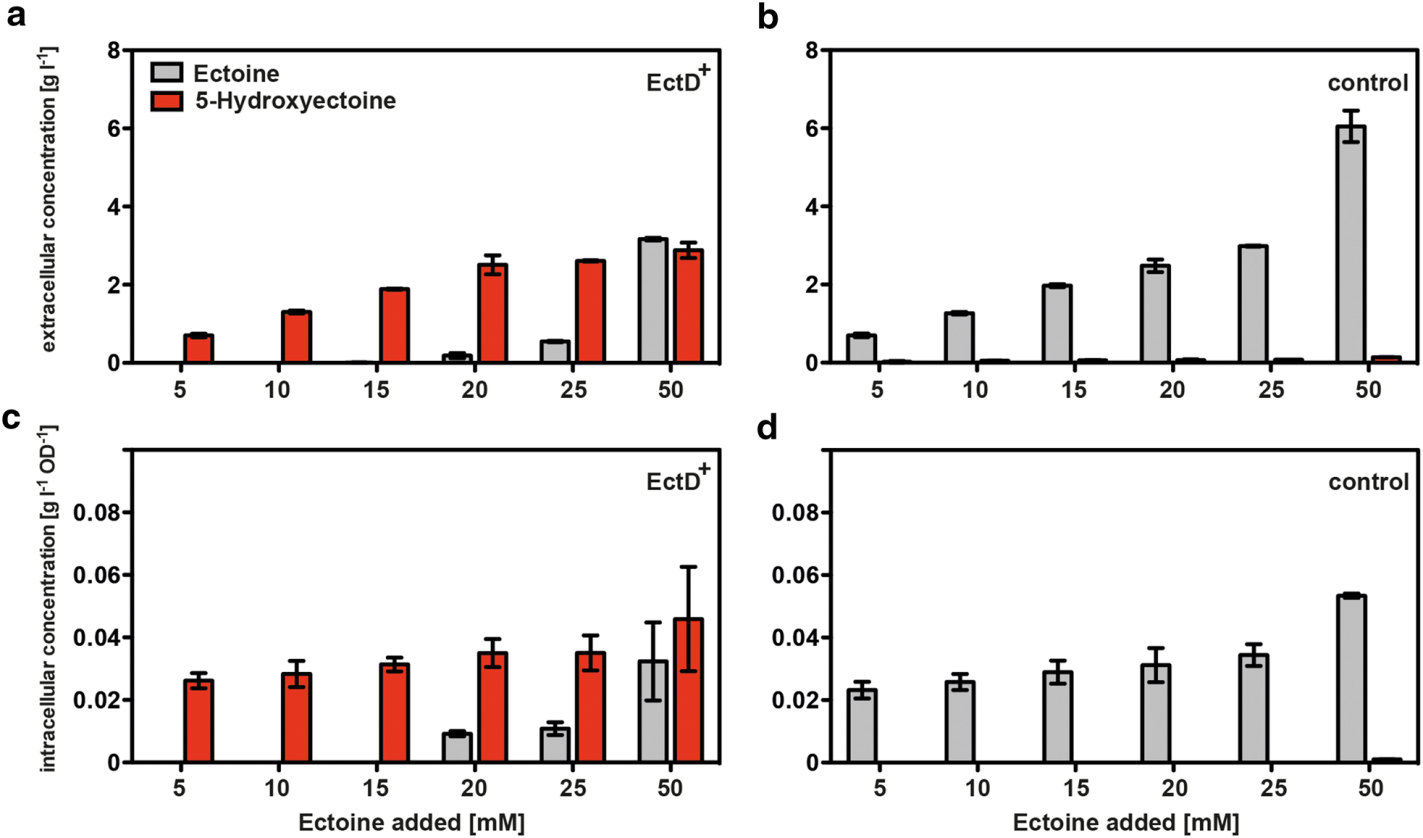
Production of 5-hydroxyectoine in response to the presence of different ectoine concentrations in the growth medium. The *E. coli* strain FF4169 containing either pMP41 (*ectD* gene from *P. stutzeri* A1501) (**a**, **c**), or the empty vector pASK-IBA3 (**b**, **d**) were grown in shake flasks containing MMA, ampicillin and 0.4 M NaCl, in the presence of various ectoine concentrations with 20 ml culture volume. After induction of *ectD* expression through the addition of AHT to the medium, the cells were further grown for 24 h, and the ectoine (*grey*) and 5-hydroxyectoine (*red*) content of the cells (**a**, **b**) or that of the supernatant (**c**, **d**) was subsequently assessed via HPLC analysis. The values shown are the means and standard deviations of two independent cultures

Another parameter that is important for the overall performance of the bioconversion of ectoine into 5-hydroxyectoine is the amount of substrate that can be added to the medium and fully converted into 5-hydroxyectoine. We therefore cultured osmotically stressed cells (with 0.4 M NaCl) in the presence of various ectoine concentrations (from 5 to 50 mM) and determined both the intracellular and extracellular pools of these compounds (Fig. 5). Up to a concentration of 15 mM ectoine (2.13 g l^−1^) in the growth medium, only 5-hydroxyectoine was found inside and outside of the cells (Fig. 5a, c). Higher concentrations of externally provided ectoine always yielded mixtures of the two ectoines (Fig. 5a, c), a situation that is not desirable for a possible practical application of the 5-hydroxyectoine producing cell factory. Control experiments with a cell factory that contained the expression vector without an insert demonstrated that there was uptake of ectoine but no conversion into 5-hydroxyectoine; hence formation of 5-hydroxyectoine was dependent on the expression of the recombinant *ectD* gene (Fig. 5b, d).

Using the information derived from experiments documented in Figs. 4a and 5a, c, we fed 15 mM ectoine to the cells and monitored its conversion into 5-hydroxyectoine over time. Growth of the cells in shake flasks for 24 h resulted in the production of an essentially ectoine-free 5-hydroxyectoine pool in the supernatant with a maximal yield of 2.3 ± 0.1 g l^−1^, a value that corresponds to 14.36 ± 0.7 mM of newly formed 5-hydroxyectoine. This conversion rate is close to that theoretically possible given that 15 mM (2.13 g l^−1^) ectoine was fed to the cells (Fig. 4b). The cell factory was also able to effectively convert different mixtures of ectoine/5-hydroxyectoine into extracellular pools that consisted only of 5-hydroxyectoine (Fig. 4b).

### Characteristics of ectoine and 5-hydroxyectoine import via the ProU and ProP transporters

**Fig. 6:**
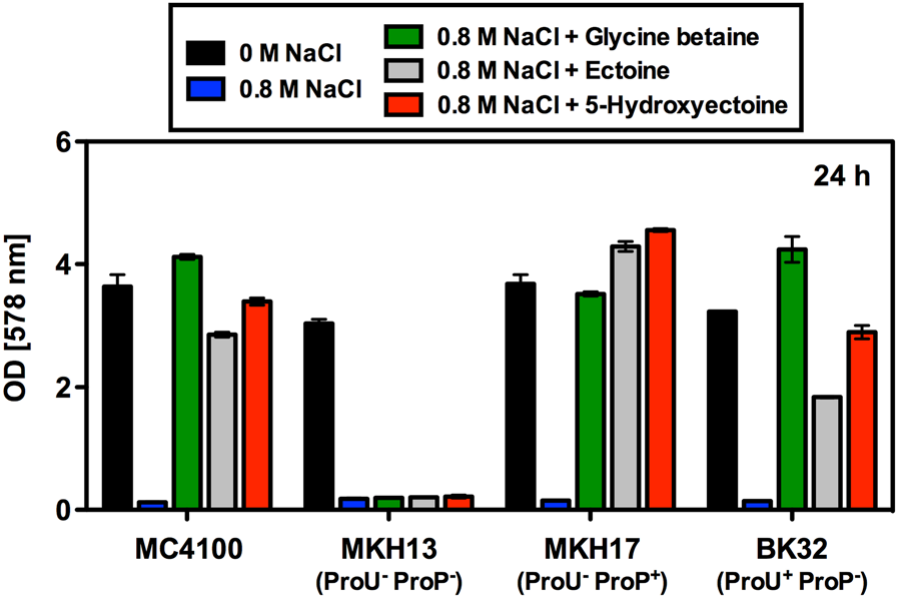
Osmostress protection by ectoine and 5-hydroxyectoine. Strains MC4100 (ProP^+^ ProU^+^), MKH13 (ProP^−^ ProU^−^), MKH17 (ProP^+^ ProU^−^) and BK32 (ProP^−^ ProU^+^) were grown either in MMA, or in MMA with 0.8 M NaCl in the absence or the presence of the osmostress protectants glycine betaine, ectoine, and 5-hydroxyectoine (provided at a final concentration of 1 mM) at 37 °C for 24 h. The obtained growth yield was determined by measuring the OD_578_ of the cultures. The values shown are the means and standard deviations of two independently grown cultures

The uptake of ectoine in *E. coli* via the ProP and ProU osmolyte uptake systems [60] is well documented [61]. In contrast, essentially nothing is known about the import of 5-hydroxyectoine, but the ProP and ProU systems are the most likely candidates. To test this, we used an isogenic set of strains in which either only ProP or ProU is functional or in which both osmolyte transporters were defective [69]. These strains were grown in a chemically defined medium (MMA) with 0.8 M NaCl, conditions under which the wild-type strain MC4100 cannot grow in the absence of an osmostress protectant (Fig. 6). The addition of either 1 mM ectoine or 1 mM 5-hydroxyectoine provided osmostress protection of the wild-type strain MC4100 and both compounds were imported via the ProP (strain MKH17) and ProU (strain BK32) systems, whereas there was no osmostress protection of strain MKH13 (Fig. 6) that is deficient in both the ProP and ProU transporters [69]. Import of ectoine and 5-hydroxyectoine via ProU provided less effective osmostress protection in comparison with a strain where these compounds were taken up via ProP (Fig. 6).

**Fig. 7:**
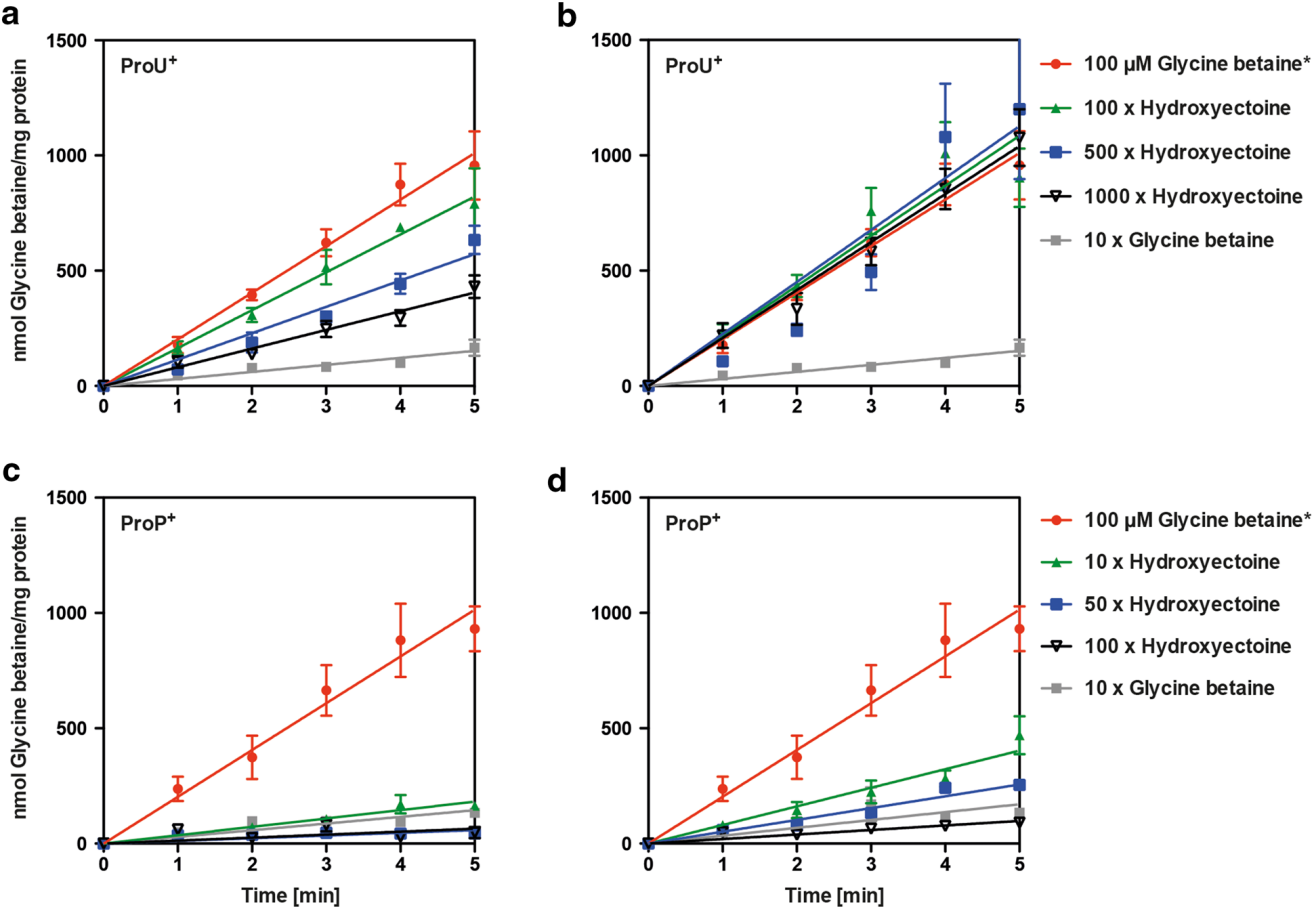
Inhibtion of the uptake of [1-^14^C]glycine betaine via the ProU and ProP transporters by an excess of ectoine and 5-hydroxyectoine. The *E. coli* mutant strains BK32 (ProP^−^ ProU^+^) (**a**, **b**) and MKH17 (ProP^+^ ProU^−^) (**c**, **d**) were cultivated in MMA with 0.4 M NaCl at 37 °C to early exponential phase (OD_578_ 0.3). Two millilitre aliquots were taken and mixed with a solution containing non-labeled glycine betaine and [1-^14^C]glycine betaine (the final concentration of glycine betaine in the uptake assay was 100 µM), and the uptake of [1-^14^C]glycine betaine by the cells was measured over time (for 5 min). Import of glycine betaine is shown in red. In parallel assays, the inhibition of [1-^14^C]glycine betaine uptake was measured with an excesses of either ectoine (**a**, **c**) or 5-hydroxyectoine (**b**, **d**). For strain BK32 (ProP^−^ ProU^+^), ectoine or 5-hydroxyectoine was provided in 100-, 500-, and 1000-fold excess; for strain MKH17 (ProP^+^ ProU^−^), ectoine or 5-hydroxyectoine was provided in 10-, 50-, and 100-fold excess. As a control, a tenfold excess of unlabeled glycine betaine was added to the [1-^14^C]glycine betaine mixture (*grey symbols*) to monitor the inhibition of [1-^14^C]glycine betaine import by glycine betaine itself. The values shown are the means and standard deviations of four independently tested cultures

To study the import of 5-hydroxyectoine and ectoine via ProP and ProU further, we conducted competition experiments with radiolabeled [^14^C]glycine betaine since this compatible solute is a major substrate for the ProU and ProP transporters and both exhibit high affinity for it [70, 71]. We measured the uptake of [^14^C]glycine betaine (provided at a final concentration of 100 μM) in the absence and presence of either ectoine or 5-hydroxyectoine as competitors and used for these experiments strains MKH17 (ProP^−^ ProU^+^) and BK32 (ProP^+^ ProU^−^). Used as a control, the addition of a tenfold excess of unlabeled glycine betaine was able to reduce [^14^C]glycine betaine import by about 85 % for both tested strains (Fig. 7).

Inhibition of [^14^C]glycine betaine import by ectoine via the ProU ABC transporter was rather weak and even a 1000-fold excess of the competitor was only able to reduce [^14^C]glycine betaine import by 45 % (Fig. 7a). Strikingly, there was essentially no competition by 5-hydroxyectoine with the uptake of [^14^C]glycine betaine via the ProU system (Fig. 7b). While osmostress protection by both ectoine and 5-hydroxyectoine (provided at a 1 mM concentration) can be observed in the long-term (24 h) growth experiment of the ProU^+^ strain MKH17 (Fig. 6), the transport assays revealed that both ectoines are not favored substrates of the ProU ABC transporter (Fig. 7a, b). This situation is different for the ProP transport system. Both ectoine and 5-hydroxyectoine competed effectively with [^14^C]glycine betaine for ProP-mediated import, with ectoine being the somewhat better competitor (Fig. 7c, d). Consequently, ProP is the physiologically more important transport system for both ectoines when they are provided at low external concentration.

### Excretion of 5-hydroxyectoine occurs independent of the MscL, MscS, and MscM mechanosensitive channels

Microbial cells employ safety valves, so called mechanosensitive channels, to prevent lysis when they are subjected to a sudden and severe osmotic down-shock [72, 73]. The opening of these cytoplasmic membrane-embedded channels is triggered by the rapid influx of water under these conditions, which in turn raises turgor and causes increased tension in the lateral plain of the membrane [51, 52, 74, 75]. Typically, microorganisms possess different types of mechanosensitive channels that possess different gating properties and channel diameters; this offers a graded response to the osmotically challenged cell. Since the structurally and functionally well characterized MscS and MscL mechanosensitive channels from *E. coli* possess large channel diameters in their fully opened forms [76, 77], their gating activity must be tightly controlled [51]. However, it cannot be firmly excluded that these channels sometimes open under osmotic steady-state conditions, and this behavior might thus be responsible for the release of 5-hydroxyectoine from the cell factory (Figs. 3, 4, 5).

We therefore investigated whether the excretion of 5-hydroxyectoine from the *E. coli* cell factory is based on the gating activity of mechanosensitive channels. For these experiments we used a set of isogenic *E. coli* strains that have previously been very carefully studied by physiological and electrophysiological approaches and which carry defects in various channel-forming proteins [72, 78]. Strain MJF465 lacks intact MscL, MscS, and MscK channels, whereas strain MJF641 is defective in all currently known mechanosensitive channels of *E. coli*, including MscM. Strain Frag 1 is the parent of these two mutant strains [79].

**Fig. 8:**
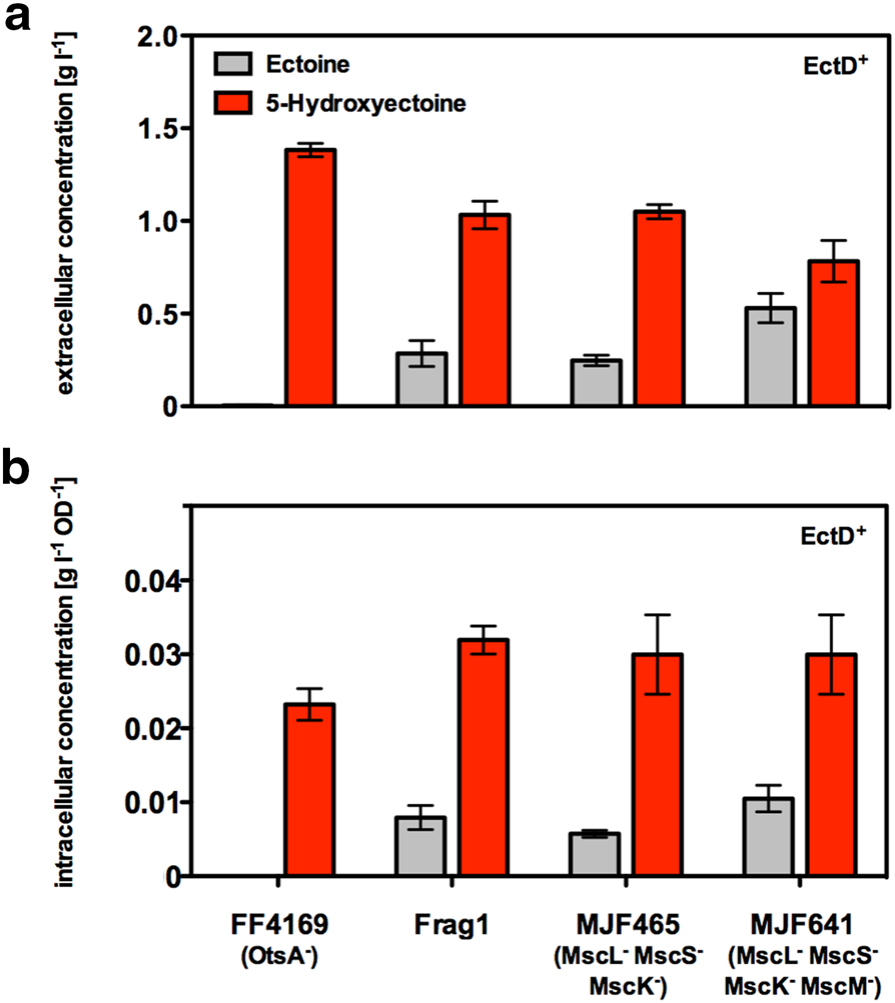
Excretion of ectoine and 5-hydroxyectoine in *E. coli* strains lacking mechanosensitive channels. The *E. coli* strains FF4169 (*otsA*::Tn*10*), Frag1 (wild-type), MJF465 (*mscL* *mscS* *mscK*), and MJF641 (*mscL* *mscS* *mscK* *mscM*) harboring the plasmid pMP41 (*ectD* gene from *P. stutzeri* A1501) were grown in MMA that contained ampicillin, 0.4 M NaCl, and 10 mM ectoine. Plasmid-based overexpression of the *ectD* gene was induced by the addition of AHT to the growth medium; the cultures were subsequently grown for 24 h. Both the extracellular (**a**) and the intracellular (**b**) concentrations of ectoine (*grey*) and 5-hydroxyectoine (*red*) were determined via HPLC analysis. The values shown are the means and standard deviations of four independent cultures. The strains with defects in various mechanosensitive channel genes (MJF465, MJF641) are derivatives of Frag1. The values shown are the means and standard deviations of four independently tested cultures

We introduced the *ectD*^+^ plasmid pMP41 into strains Frag1, MJF465, and MJF641, and then exposed them to sustained high-salinity growth conditions (by adding 0.4 M NaCl to MMA) in the presence of 10 mM ectoine. After growth for 24 h, we then measured the ectoine and 5-hydroxyectoine pools in the supernatants of the cultures. In all three strains, large amounts of 5-hydroxyectoine were found in the supernatant (Fig. 8a). Since this is also the case in strain MJF641 lacking all currently characterized mechanosensitive channels (MscL, MscS, MscK, MscM), we can firmly conclude that the release of the newly formed 5-hydroxyectoine from the cell factory does not occur via the transient opening of these types of channels under osmotic steady-state conditions.

In direct comparison with the cell factory strain FF4169 (*otsA*::Tn*10*) carrying the same *ectD*^+^ plasmid pMP41, Frag 1 and its channel-mutant derivatives strains MJF465 and MJF641, the supernatant of the cultures still contained substantial amounts of the precursor ectoine (Fig. 8a). This could potentially be explained by inefficient ectoine import or an ineffective biotransformation of ectoine to 5-hydroxyectoine. The sum of the measured ectoine and 5-hydroxyectoine concentrations in the supernatant of strain MJF641 was 1.31 ± 0.4 g l^−1^ compared to the 5-hydroxyectoine content of 1.38 ± 0.1 g l^−1^ found in strain FF4169 (Fig. 8a). Hence, like strain FF4169, the channel-deficient strains MJF465 and MJF641 and their parent Frag1 do not permanently accumulate massive amounts of ectoines in their cytoplasm under the growth conditions that we have used for our cell factory (Fig. 8b).

## Discussion

Whole cell biocatalysts become increasingly important for environmentally friendly and resource-preserving production of compounds with biotechnological and medical interest. They also provide a suitable chemical space for the regeneration of co-factors and co-substrates required for the functioning of enzymes [80], a process on which the ectoine hydroxylase is dependent [26, 27, 29–31]. As a member of the non-heme-containing iron(II) and 2-oxoglutarate-dependent dioxygenase superfamily [32–35], the ectoine hydroxylase relies on a mononuclear iron center and uses 2-oxoglutarate as its co-substrate [26, 27, 29–31]. The region- and stereo-specific hydroxylation of chemically non-activated carbon atoms is often difficult to achieve through organo-chemical synthesis. However, the ectoine hydroxylase performs such am enzymatic reaction with high precision and efficiency, both in vitro and in vivo [20, 26, 81]. It converts (4*S*)-ectoine into (4*S*,5*S*)-5-hydroxyectoine in a single step [26, 27, 29, 30].

The function-preserving attributes of ectoines have led to various practical applications and the development of a biotechnological industrial-scale production process [45, 48, 49]. This *bacterial milking* procedure [53] relies on the transient opening of mechanosensitive channels [51, 52] to release the synthesized ectoines from the producer cells in response to a severe osmotic down-shock [48]. Here, we report on the properties of an *E. coli*-based synthetic cell factory that releases almost all of the newly formed 5-hydroxyectoine into the medium under osmotic steady-state growth conditions (Figs. 3c, 4, 5a, 8a). Most interestingly, we found that the excretion of 5-hydroxyectoine from the recombinant cell factory occurs independently of all currently known mechanosensitive channels operating in *E. coli* (MscL, MscS, MscK, MscM) [78].

Excretion of ectoine from recombinant *E. coli* cells expressing the *ectABC* biosynthetic genes has been observed before [55, 57], but the underlying mechanism has remained unclear. Schubert et al. [57] found that the release of ectoine was not accompanied by the accumulation of amino acids in the growth medium, a consequence expected from the transient opening of mechanosensitive channels [51, 52]. These authors therefore argued that ectoine had been released in their experiments via a specific solute efflux system [57]. It is well known that microorganisms possess efflux systems for various types of compounds, in particular for amino acids [82]. One might therefore ask what physiological function and advantage a compatible solute efflux system would offer in comparison with the functioning of mechanosensitive channels. Due to the large diameter of MscS- and MscL-type channels in their open forms (about 13 and 30 Å, respectively) [74–77], these safety valves will indiscriminately release all low-molecular weight compounds from the cell upon a sudden osmotic downshift, a process that negatively impinges on cell growth [51, 52]. An efflux system dedicated to the excretion of compatible solutes would avoid the loss of valuable metabolites. It would also permit the cell to respond more specifically to imbalances in turgor that temporarily might arise during cell elongation and division [83]. A dedicated efflux system could also release compatible solutes under steady-state osmotic conditions when the need arises, whereas the opening of mechanosensitive channels requires an osmotic downshift [51, 52].

Although the molecular identity of such putative compatible solute efflux systems in microorganisms has remained tenuous [84], several observations point to their existence. The release of glycine betaine from osmotically down-shocked *Lactobacillus plantarum* cells revealed two kinetically distinguishable components; one is related to the operation of mechanosensitive channels, and the other is consistent with a carrier-mediated export process [85]. Furthermore, in different microbial species, substantial accumulation of newly synthesized compatible solutes in the growth medium occurs when the major uptake system for these osmoprotectants is genetically inactivated [83, 86–89]. Particular relevant for our study is the report of Grammann et al. [86], who found considerable amounts of newly synthesized ectoine in the supernatant of an *H. elongata* mutant deficient in the ectoine/5-hydroxyectoine uptake system TeaABC. Furthermore, Jebbar et al. [61] observed a prompt efflux of prior imported radiolabeled ectoine in *E. coli*, when unlabeled ectoine was added to the growth medium. While it is still unclear which system(s) is responsible for the excretion of compatible solutes from the producer cells, this phenomenon worked to our advantage to recover the recombinantely produced 5-hydroxyectoine from our cell factory in the supernatant.

The seven ectoine hydroxylases whose performance we have evaluated in the context of the set-up of our cell factory are closely related in their amino acid sequences (Additional file 1) and those residues that are of functional importance (Fig. 1a) [22, 27] are all strictly conserved (Additional file 2). These EctD-type enzymes exhibit similar kinetic characteristics in vitro (Additional file 1) when they were assayed under conditions optimized for each of them [21, 22]. To our surprise, we observed substantial differences in the efficiency of the biotransformation of ectoine to 5-hydroxyectoine, when we benchmarked the various EctD enzymes in vivo against each other (Fig. 3c). While differences in the expression level of *ectD* or the stability of the produced EctD enzymes in the heterologous *E. coli* host strain might be contributing factors (Fig. 3a, b), other characteristics of these ectoine hydroxylases must come into play to explain their different performances. These differences might be related to the ion pools of the *E. coli* cytoplasm since small but noticeable variations in response to salts have been reported in the course of the biochemical in vitro characterization of various EctD enzymes [21, 22].

When we fed 15 mM ectoine to the *E. coli* cell factory expressing the *P. stutzeri* A1501 *ectD* gene, approximately 98 % of the newly formed 5-hydroxyectoine was found in the supernatant after 24 h of growth. Under these conditions, there is essentially no contaminating ectoine left in the growth medium and the missing 2 % of 5-hydroxyectoine is found inside the cells (Fig. 5a, c). With notable exceptions, most microorganisms that can produce both ectoine and 5-hydroxyectoine contain a varied mixture of these compounds [21, 24, 26, 36, 37, 39]. Such mixtures of the two ectoines, either extracted or released from the natural producer cells via an osmotic down-shock, can be effectively converted into essentially ectoine-free 5-hydroxyectoine solutions by our synthetic cell factory (Fig. 4c).

The uptake of ectoine through the promiscuous compatible solute importer systems ProP and ProU has previously been assessed [61, 71], but the import characteristics of 5-hydroxyectoine had not been studied in any detail. Our data show that osmostress protection of *E. coli* by either ectoine or 5-hydroxyectoine can be observed in long-term growth experiments with a ProU^+^ ProP^−^ strain (Fig. 6). However, competition transport experiments with radiolabel glycine betaine showed that both ectoine and 5-hydroxyectoine are not favorable substrates for ProU (Fig. 7a, b). This can readily be understood in view of the architecture of the ligand-binding site present in the periplasmic solute receptor protein ProX operating in conjunction with the ProU ABC transporter [60, 70]. Its aromatic cage is designed for the efficient capturing of compounds possessing either tri- (e.g. glycine betaine), or di- (e.g. proline betaine)-methlyammonium head-groups [69, 90]. As a consequence, the ligand-binding site of ProX is not optimal for the binding of ectoines when one considers the architectural features of the substrate-binding sites of true high-affinity ligand-binding proteins for these types of solutes; e.g., EhuB, UehA, and TeaA [91–93].

In contrast to ProU, the osmotically stimulated ProP transporter [62] exhibits reasonable affinities for both ectoine and 5-hydroxyectoine in competition assays with glycine betaine (Fig. 7c, d). Hence, this member of the MFS superfamily with its broad substrate specificity [71] is certainly the dominant ectoine and 5-hydroxyectoine transporter of *E. coli*. This conclusion is fully consistent with previously reported measurements of ectoine pools built up by osmotically stressed (with 0.7 M NaCl) *E. coli* cells via import, either via the ProP or the ProU systems [61].

Although our cell factory can import 5-hydroxyectoine under osmotic stress conditions, the cells retain only very modest amounts of it when it is newly formed from ectoine via EctD (Fig. 5a, c). These amounts are apparently sufficient to physiologically cope with the sustained but moderate osmotic stress that we imposed onto the *E. coli* cells. Hence in terms of practical application, the re-import of originally excreted 5-hydroxyectoine is apparently of no great concern for the overall performance of our synthetic cell factory.

Synthetic ectoine derivatives with reduced or expanded ring sizes have been reported and shown to possess attributes different from those of ectoine, for instance in their performance as PCR enhancers [43]. The hydroxylation of such synthetic ectoines (or compounds chemically related to ectoine) might endow them with novel and beneficial characteristics, as has been found in connection with studies assessing 5-hydroxyectoine for its superior desiccation stress and membrane-protecting potential [16, 40–42]. Realizing this, a patent envisioning the import of ectoine-related compounds via osmotically inducible transport systems, their hydroxylation via EctD, and the active or passive release of the newly formed compounds into the growth medium has been granted [94]. However, claims made in this patent have not yet been subjected to scientific scrutiny via a peer-reviewed publication.

The efficiency by which compatible solute transport systems such as ProP and ProU [60, 62] (Figs. 6, 7) might import synthetic ectoine-related substrates will be a key factor in realizing the full potential of cell factories for these compounds. The evaluation of the in vivo performance of a considerable number of ectoine hydroxylases for their natural substrate carried out in this study (Fig. 3) strongly hints that not any arbitrary EctD protein will be optimally suited for the hydroxylation of synthetic ectoine derivatives. Of similar importance is the affinity of the chosen EctD protein for synthetic ectoines and for the proper positioning of these non-natural substrates within the active site of the ectoine hydroxylase (Fig. 1a) to allow a position and stereo-specific hydroxylation reaction with high efficiency and precision [27].

## Conclusions

We report here the basic design and functional characterization of a synthetic microbial cell factory that can execute the position- and stereo-specific hydroxylation of externally provided ectoine through the activity of the ectoine hydroxylase [26, 27, 29]. An important result of our study is the observation that ectoine hydroxylases possessing similar in vitro enzyme characteristics (Additional file 1) can perform quite differently when produced in a heterologous chassis strain. The 5-hydroxyectoine formed in our synthetic cell factory is almost quantitatively excreted into the growth medium from which it can be readily purified [48]. Excretion occurs in a manner that is independent of all currently known mechanosensitive channels of *E. coli* [78], a finding that points to the existence of a compatible solute efflux system in this microorganism. The architecture of the active site of the ectoine hydroxylase, as revealed by crystallographic analysis (Fig. 1a) [27], is probably flexible enough to allow the hydroxylation of already reported synthetic ectoines with slightly reduced or expanded ring sizes [43]. By carefully considering the structural design and spatial constraints of the ectoine hydroxylase catalytic core (Fig. 1a) [27], it might be possible to rationally devise in silico new ectoine derivatives optimized for their chemical modification via EctD. Taken this knowledge together, the cell factory reported here might find biotechnologically interesting applications in chemical biology.

## Methods

### Chemicals and reagents

Ectoine and 5-hydroxyectoine were kindly provided by the bitop AG (Witten, Germany). Anhydrotetracycline-hydrochloride (AHT) was purchased from IBA GmbH (Göttingen, Germany). Acetonitrile (HPLC-grade) was obtained from VWR International GmbH (Darmstadt, Germany). Ampicillin and all other chemicals were purchased from Serva Electrophoresis GmbH (Heidelberg, Germany) and Carl Roth GmbH (Karlsruhe, Germany). Radiolabeled [1-^14^C]glycine betaine (55 mCi mmol^−1^) was bought from American Radiolabeled Chemicals Inc. (St. Louis, MO; USA).

### Bacterial strains and plasmids

The *E. coli* strain FF4169 (*otsA::Tn10*)1 is deficient in the synthesis of trehalose [66]. It is a derivative of strain MC4100 [95]. This latter *E. coli* strain is also the parent of strains BK32 [Δ(*proP*)2 *proU*^+^], MKH17 [*proP*^+^ Δ(*proU*::*spc*)608 (Spc^r^)], and MKH13 [Δ(*proP*)2 Δ(*proU*::*spc*)608] (Spc^r^)] carrying in various combinations defects in the genes encoding the ProP or ProU compatible solute uptake systems [60, 69]. To assess a possible contribution of mechanosensitive channels to the release of 5-hydroxyectoine from the recombinant *E. coli* cell factory, we used strains Frag1, MJF465, and MJF641; these strains have the following genotypes. Frag1: (F^−^, *rha, gal, thi, lac*); MJF465 (FRAG1, *mscL::Cm;* Δ*mscS; mscK::Kan*), and MJF641 (FRAG1, *mscL, mscS, mscK, ybdG, ybiO, yjeP, ynaI*) [72, 78, 79].

The construction of the expression plasmids containing the ectoine hydroxylase structural gene (*ectD*) [26] from various *Bacteria* and *Archaea* has been described [21, 22, 26]. We used the following plasmids: pBJ10 (EctD from *V.salexigens*; accession number: AY935522), pMP32 (EctD from *H.elongata*; accession number: WP_013333764.1), pMP40 (EctD from *S.alaskensis*; accession number: WP_011543221.1), pMP41 (EctD from *P.stutzeri*; accession number: ABP77885.1), pMP43 (EctD from *P.lautus*; accession number: AER00258.1), pMP44 (EctD from *A.ehrlichii*; accession number: AER00257.1), and pMP45 (EctD from *N.maritimus*; accession number: AER00259.1) [21, 22, 26]. The transcription of these various plasmid-encoded *ectD* genes is mediated by the *tet* promoter present on the backbone of the expression vectors pASG-IBA3 and pASK-IBA3 (IBA GmbH, Göttingen) used for the construction of the *ectD* expression plasmids [21, 22, 26]. The *tet* promoter is controlled by the TetR repressor and its transcriptional activity can be induced by adding AHT to the growth medium.

### Growth media for *E. coli* strains

All *E. coli* strains were routinely maintained on Luria Bertani (LB) agar plates and propagated in liquid LB medium [96]. When they contained a recombinant plasmid, ampicillin (100 µg ml^−1^) was added to the growth medium. For experiments involving the bioconversion of ectoine to 5-hydroxyectoine, *E. coli* strains were grown in minimal medium A (MMA) [96] supplemented with 0.5 % (w/v) glucose as the carbon source, 1 mM MgSO_4_, and 3 mM thiamine. The osmolarity of the growth medium was adjusted by adding various concentrations of NaCl, as specified in the individual experiments. Shake-flask cultures were incubated at 37 °C in a shaking water bath set to 220 rpm. Osmostress protection assays with *E. coli* strains were conducted in 100-ml Erlenmeyer flasks (culture volume of 20 ml) by growing the cells (at 37 °C) in MMA containing 0.8 M NaCl in absence or presence (1 mM final concentration) of the tested compatible solutes [69]. The growth yield of these cultures was recorded after 24 h by measuring their OD_578_.

### Biotransformation of ectoine into 5-hydroxyectoine in shake flasks

*Escherichia coli* cells harboring an *ectD*^+^ plasmid, were inoculated into LB medium and incubated for 5 h on a roller at 37 °C. Two hundred microlitre of this culture were then transferred into 20 ml MMA and incubated in a shaking water bath overnight (set to 220 rpm, 37 °C). This pre-culture was used to inoculate fresh media (to an OD_578_ of 0.1) (20 ml MMA) containing various amounts of NaCl and ectoine. When these main cultures contained more than 0.5 M NaCl, cells from the pre-culture were pre-adapted in MMA containing 0.3 M NaCl. All main cultures were grown until they reached an OD_578_ of 0.5. At this time, AHT was added to a final concentration of 0.2 mg l^−1^ to induce the expression of the plasmid-encoded *ectD*^+^ genes; the cells were then grown for additional 24 h. Two times 2-ml samples of each culture were harvested by centrifugation (16,000*g*, for 10 min at room temperature); 1 ml of the supernatant and the cell pellet were stored at −80 °C until further use.

The production of recombinant EctD proteins in these cells was analyzed by SDS polyacrylamide gel electrophoresis [97]. For Western blot analysis of these samples, cell pellets were re-suspended to an OD_578_ of ten in TE buffer (10 mM Tris–HCl, pH 7.5, 1 mM EDTA) with lysozyme (final concentration: 1 mg ml^−1^). Fifty microlitre portions of these samples were incubated for 5 min at 37 °C, mixed with 25 µl SDS-PAGE sample buffer, and subsequently incubated for 5 min at 95 °C. After centrifugation in an Eppendorf table-top centrifuge (10 min, 16,000*g*), 10 µl portions of the samples were applied to an 15 % SDS–polyacrylamide gel; the electrophoretically separated proteins were then transferred to a polyvinylidenfluoride (PVDF) membrane via semi-dry blotting. Western blotting of the transferred proteins was performed with a primary mouse monoclonal anti *Strep*-tag II (SA-WSHPQFEK) antibody (purchased from IBA GmbH; Göttingen, Germany), and the formed immune complex was detected with a secondary rabbit anti-mouse alkaline phosphatase-coupled IgG antibody (purchased from Promega, Madison, WI, USA) and the CDP-Star Western blotting detection reagent (Roche Diagnostics GmbH, Mannheim, Germany). Signals were detected by chemiluminescence using an imager system (ChemoCam Imager, Intas Science Imaging Instruments GmbH, Göttingen, Germany).

### HPLC analysis of ectoine and 5-hydroxyectoine

Cell pellets of *E. coli* strains were either lyophilized for the determination of the dry weight, or low-molecular-weight compounds were extracted with 70 % ethanol. For this purpose, the cell pellets were re-suspended in 1-ml 70 % ethanol and were shaken for 1 h. After centrifugation at 16,000*g* (4 °C, 30 min) to remove cell debris, the ethanolic extracts were transferred into fresh Eppendorf tubes, and the ethanol was removed by evaporation (at 55 °C for 20 h). The resulting dried material was suspended in 100 µl of distilled water and insoluble material was removed by centrifugation (16,000*g* at 4 °C for 30 min). The extracted samples and the cell-free culture supernatant were diluted tenfold with distilled water and acetonitrile (the end concentration of acetonitrile was 50 %) and analyzed for their ectoine/5-hydroxyectoine content by isocratic high-performance liquid chromatography (HPLC) [98]. For these measurements we employed an Agilent 1260 Infinity LC system (Agilent, Waldbronn, Germany) and a GROM-SIL Amino 1PR column (GROM, Rottenburg-Hailfingen, Germany) essentially as described [98] with the exception that a 1260 Infinity Diode Array Detector (DAD) (Agilent) was used, instead of the previously used UV/Vis detector system. The ectoine content of samples was quantified using the OpenLAB software suite (Agilent). Standard curves for the calculation of the ectoine and 5-hydroxyectoine concentrations were determined with commercially available samples (obtained from bitop AG, Witten, Germany).

### Transport studies with radiolabeled glycine betaine

To determine glycine betaine uptake and its inhibition by ectoine and 5-hydroxyectoine in cultures of the *E. coli* strain MKH17 (ProP^+^ ProU^−^) and BK32 (ProP^−^ ProU^+^), cells were grown in MMA containing 0.4 M NaCl to an OD_578_ of 0.3 at 37 °C. Uptake of [1-^14^C]glycine betaine by the cells was assayed at various time points in 2-ml aliquots at 37 °C with a final glycine betaine concentration of 100 µM. The inhibition of [1-^14^C]glycine betaine uptake by ectoine and 5-hydroxyectoine was measured by adding 10-, 50- and 100-fold excesses of the ectoine and 5-hydroxyectoine competitors in case of cells from strain MKH17 (ProP^+^ ProU^−^), and 100-, 500- and 1000-fold excesses of the competitors in case of the cells from strain BK32 (ProP^−^ ProU^+^). Import of radiolabeled [1-^14^C]glycine betaine was followed for 5 min and the amount of [1-^14^C]glycine betaine taken up by the *E. coli* cells was determined by scintillation counting. The assay conditions followed a previously described protocol [99].

### Alignment of EctD proteins and in silico modeling of the ectoine hydroxylase from *P. stutzeri*

Amino acid sequences of the studied EctD proteins were retrieved from the NCBI database (http://www.ncbi.nlm.nih.gov/) and aligned using Clustal Omega (http://www.ebi.ac.uk/Tools/msa/clustalo/) [100]. A structural model of the EctD protein from *P. stutzeri* A1501 was built via the SWISS model web server (https://swissmodel.expasy.org/) [101] and is based on the crystal structure of the ectoine hydroxylase from *S. alaskensis* (PDB ID: 4Q5O) [27]. This model had an overall Global Model Quality Estimation score of 0.76, a ranking where a score of 1 indicates perfect identity of the experimentally determined crystal structure of the reference protein and the derived in silico model of the target protein [101]. Structures of EctD proteins were visualized and analyzed using the PyMOL Molecular Graphics System suit (https://www.pymol.org) [102].

## Additional files

10.1186/s12934-016-0525-4 Characteristics of the studied ectoine hydroxylases. The data shown were compiled from previous publications [21, 22].
10.1186/s12934-016-0525-4 Amino acid sequence alignment of selected EctD proteins. The EctD amino acid sequences from *V. salexigens*, *H. elongata, S. alaskensis, P. lautus, A. ehrlichii*, and *N. maritimus* were aligned using the ectoine hydroxylase from *P. stutzeri* A1501 as the query template. The residues of the ectoine hydroxylase coordinating the iron catalyst are highlighted in red, those that bind the co-substrate 2-oxoglutarate are marked in blue, and the residues involved in the binding of ectoine/5-hydroxyectoine are depicted in green [27]. The consensus sequence for ectoine hydroxylases [22, 27, 31] is highlighted in yellow.

## Abbreviations

AHT: anhydrotetracycline-hydrochloride
LB: Luria–Bertani medium
MMA: minimal medium A
PCR: polymerase chain reaction
rpm: revolutions per minute
SDS: sodium-dodecylsulfate
Spc^r^: spectinomycin resistance
