# Supplementary material for: EctD-mediated biotransformation of the chemical chaperone ectoine into hydroxyectoine and its mechanosensitive channel-independent excretion
Source: Microb Cell Fact. 2016 Jul 20;15:126. doi: 10.1186/s12934-016-0525-4 (PMC4955205; doi:10.1186/s12934-016-0525-4)
Supplement: Supplementary file 1 — 10.1186/s12934-016-0525-4 Characteristics of the studied ectoine hydroxylases. The data shown were compiled from previous publications [21, 22]. [file 12934_2016_525_MOESM1_ESM.docx]

**Table. S1** Characteristics of the studied ectoine hydroxylases.

| Organism | sequence identity^1^ [%] | MW  [kDa] | length  [AA] | pI | *K*_m_  [mM ectoine] | *V*_max_  [U/mg] | *k*_cat_  [s^-1^] | Accession number | Reference |
| --- | --- | --- | --- | --- | --- | --- | --- | --- | --- |
| *P. stutzeri* | 100 | 34.2 | 302 | 5.5 | 6.2 ± 0.4 | 6.7 ± 0.2 | 3.6 | ABP77885.1 | [1] |
| *S. alaskensis* | 52.8 | 34.1 | 306 | 5.5 | 9.8 ± 0.5 | 1.0 ± 0.2 | 1.7 | WP_011543221.1 | [1] |
| *V. salexigens* | 49.8 | 34.4 | 300 | 5.8 | 5.9 ± 0.3 | 6.4 ± 0.2 | 3.2 | AY935522 | [1] |
| *N. maritimus* | 47.4 | 34.7 | 304 | 5.8 | 3.8 ± 0.5 | 1.8 ± 0.1 | 1.0 | AER00259.1 | [2] |
| *P. lautus* | 46.6 | 34.8 | 302 | 5.6 | 9.5 ± 0.7 | 1.3 ± 0.1 | 0.6 | AER00258.1 | [1] |
| *A. ehrlichii* | 41.4 | 34.3 | 302 | 5.7 | 9.0 ± 0.3 | 1.0 ± 0.1 | 0.7 | AER00257.1 | [1] |
| *H. elongata* | 40.9 | 37.4 | 332 | 5.8 | 5.7 ± 0.6 | 2.5 ± 0.2 | 1.0 | WP_013333764.1 | [1] |

^1^Amino acid sequences was aligned and compared to the protein sequence of the *P. stutzeri* A1501 ectoine hydroxylase. Hence, the degree of amino acid sequence identity of the various EctD proteins is given relative to that of the *P. stutzeri* A1501 EctD enzyme.

The listed data were compiled from the following publications:

[1] Widderich N, Höppner A, Pittelkow M, Heider J, Smits SH, Bremer E: Biochemical properties of ectoine hydroxylases from extremophiles and their wider taxonomic distribution among microorganisms. PLoS One*.* 2014; 9:e93809.

[2] Widderich N, Czech L, Elling FJ, Könneke M, Stöveken N, Pittelkow M, Riclea R, Dickschat JS, Heider J, Bremer E: Strangers in the archaeal world: osmostress-responsive biosynthesis of ectoine and hydroxyectoine by the marine thaumarchaeon *Nitrosopumilus maritimus*. Env Microbiol*.* 2016; 18:1227-1248.
