# Supplementary figures and images for: EctD-mediated biotransformation of the chemical chaperone ectoine into hydroxyectoine and its mechanosensitive channel-independent excretion
Source: Microb Cell Fact. 2016 Jul 20;15:126. doi: 10.1186/s12934-016-0525-4 (PMC4955205; doi:10.1186/s12934-016-0525-4)

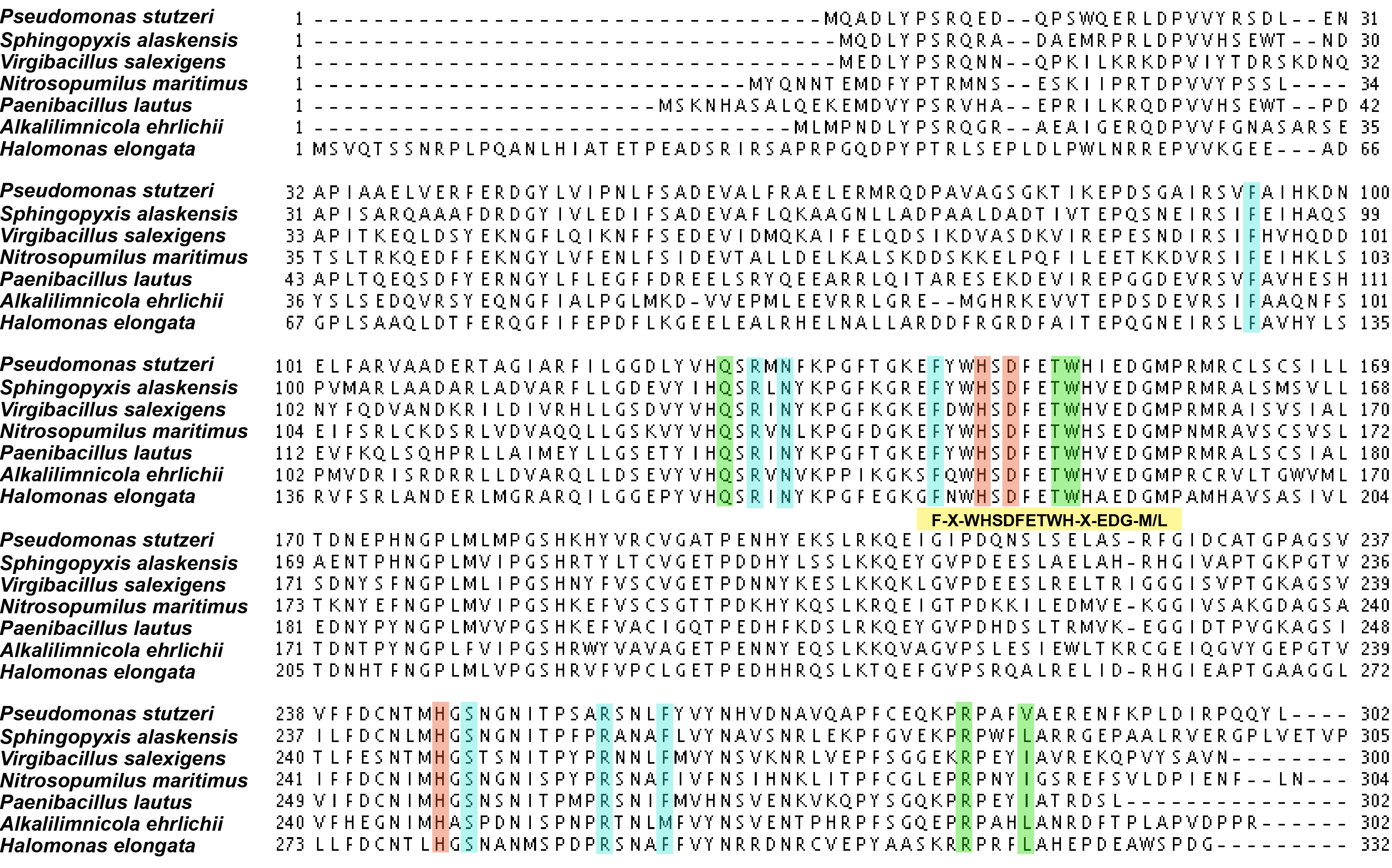

Supplement: Supplementary file 2 — 10.1186/s12934-016-0525-4 Amino acid sequence alignment of selected EctD proteins. The EctD amino acid sequences from V. salexigens, H. elongata, S. alaskensis, P. lautus, A. ehrlichii, and N. maritimus were aligned using the ectoine hydroxylase from P. stutzeri A1501 as the query template. The residues of the ectoine hydroxylase coordinating the iron catalyst are highlighted in red, those that bind the co-substrate 2-oxoglutarate are marked in blue, and the residues involved in the binding of ectoine/5-hydroxyectoine are depicted in green [27]. The consensus sequence for ectoine hydroxylases [22, 27, 31] is highlighted in yellow. [file 12934_2016_525_MOESM2_ESM.tif]
